# The Effects of Graphene-Family Nanomaterials on Plant Growth: A Review

**DOI:** 10.3390/nano12060936

**Published:** 2022-03-12

**Authors:** Xiao Zhang, Huifen Cao, Haiyan Wang, Jianguo Zhao, Kun Gao, Jun Qiao, Jingwei Li, Sai Ge

**Affiliations:** 1 Key Laboratory of National Forest and Grass Administration for the Application of Graphene in Forestry, Institute of Carbon Materials Science, Shanxi Datong University, Datong 037009, China; xiaozhang@sxdtdx.edu.cn (X.Z.); jgzhaoshi@126.com (J.Z.); qiaojun_nk@163.com (J.Q.); bjyddx1988@163.com (J.L.); gs_sxdtdx@163.com (S.G.); 2 College of Agriculture and Life Science, Shanxi Datong University, Datong 037009, China; gaokunnew@aliyun.com; 3 College of Chemistry and Chemical Engineering, Shanxi Datong University, Datong 037009, China

**Keywords:** graphene-family nanomaterials, plants growth, fate, morphological effects, physiological and biochemical effects, cytological effects, differentially expressed genes

## Abstract

Numerous reports of graphene-family nanomaterials (GFNs) promoting plant growth have opened up a wide range of promising potential applications in agroforestry. However, several toxicity studies have raised growing concerns about the biosafety of GFNs. Although these studies have provided clues about the role of GFNs from different perspectives (such as plant physiology, biochemistry, cytology, and molecular biology), the mechanisms by which GFNs affect plant growth remain poorly understood. In particular, a systematic collection of data regarding differentially expressed genes in response to GFN treatment has not been conducted. We summarize here the fate and biological effects of GFNs in plants. We propose that soil environments may be conducive to the positive effects of GFNs but may be detrimental to the absorption of GFNs. Alterations in plant physiology, biochemistry, cytological structure, and gene expression in response to GFN treatment are discussed. Coincidentally, many changes from the morphological to biochemical scales, which are caused by GFNs treatment, such as affecting root growth, disrupting cell membrane structure, and altering antioxidant systems and hormone concentrations, can all be mapped to gene expression level. This review provides a comprehensive understanding of the effects of GFNs on plant growth to promote their safe and efficient use.

## 1. Introduction

Graphene is a two-dimensional nanomaterial composed of one or more layers of carbon atoms with different modifications possible at the edges. Graphene was first discovered by Andre Geim and Constantine Novoselov in 2004 [1], for which they were awarded the Nobel Prize in Physics in 2010. Graphene-family nanomaterials (GFNs) can be divided into several categories based on the number of layers, lateral dimensions, surface chemistry, defect density, and composition; the categories include few-layer graphene (FLG), ultrathin graphite, graphene oxide (GO), reduced graphene oxide (RGO), graphene quantum dots (GQD), sulfonated graphene oxide (SGO), amine-functionalized graphene oxide (G-NH_2_), and hydrated graphene ribbon (HGR) [2,3]. Although members of each group have properties in common, such as a huge specific surface area, excellent mechanical properties, and superior electrical conductivity [4], they also have their own unique properties and functions.

The past two decades have witnessed an increase in the application of graphene in fields such as electronics [5], biomedicine [6], energy storage [7], environmental protection [8], and agriculture [9]. With the growing use of graphene materials, there are parallel public concerns about the safety of their release into the environment [10]. In contrast to the long-term and in-depth research that has been conducted into GFN exposure in animals [2,11,12,13,14,15], studies into the effects of GFNs on plants are just beginning.

Although there have been a large number of experiments related to the bioeffects of GFNs, the results of different studies are inconsistent, which has hindered the use of GFNs in the fields of agriculture and forestry. There are many reviews related to GFN–plant interactions that discuss the application prospects of GFNs [16,17,18,19,20]. Due to limitations in the depth of published research, the existing reviews summarize the morphological, cytological, physiological, and biochemical effects [10,16,19,21,22,23,24] without addressing alterations in gene expression. In this review, we summarize the fate of GFNs in plants and the effects of GFNs on plant growth phenotypes from the morphological to molecular scales. This review presents a comprehensive picture of the bioeffects of GFNs to pave the way for more efficient use.

## 2. The Fate of GFNs in Plants

The uptake, accumulation, translocation, and metabolism of graphene in plants is not only a fundamental scientific issue but also critical to understand in terms of its environmental risks and biosafety. However, carbon is the most basic element that makes up living organisms; plants themselves contain large amounts of carbon, and GFNs cannot be quantified by atomic emission spectroscopy, while many metal-containing nanoparticles can [25]. At present, the absorption and intracellular localization of GFNs in plants are mainly studied by probe labeling [25,26,27], transmission electron microscopy (TEM), and Raman spectra [28,29,30,31,32].

Although there are some technical requirements in material preparation, probe labeling has attracted much attention due to its high sensitivity [33]. Chen et al. found that ^13^C-labelled GFNs were translocated from roots into leaves in wheat (^13^C-GO) and pea plants (^13^C-GO and ^13^C-RGO) [25,27]. Wang et al. also found that Rho-B labelled GO could be translocated from roots to leaves [34]. Huang et al. observed that ^14^C labeled FLG could be absorbed by the roots and transported to the shoots. ^14^C-FLG was found to penetrate cell walls and membranes into the chloroplasts, but no ^14^C-FLG was detected in seeds. The ^14^C-FLG transported to the shoot was eventually degraded into ^14^CO_2_ [26].

Using transmission electron microscopy, the presence or absence of GFNs in different tissues can be easily detected. Graphene deposited on seed surfaces can penetrate seed husks at the germination stage [30]. Similarly, Vochita et al. observed graphene accumulation in wheat germ during early ontogenetic stages [29]. Graphene has also been identified inside root tip cells at the seedling growth stage [30]. GO accumulation was observed in root hair and root parenchyma cells but not in the leaf, stem, or root sieve element [28]. Hu et al. revealed that graphene was transferred from wheat roots to shoots and was found in the cytoplasm and chloroplasts [32]. In TEM images, GFN accumulations appear as black dots or bands. In many studies, the properties of these substances can be further identified using Raman spectroscopy [30,32,35], which is a simple, fast, and nondestructive detection technique [36]. The D band (located near 1350 cm^−1^) and G band (located near 1580 cm^−1^) can be used as the two main representative Raman peaks for GFNs [37,38].

It can be concluded from the above research that GFNs penetrate seed husks and enter germs at the germination stage; GFNs can be absorbed by the root system and transported to above-ground tissues at the seedling growth stage; and GFNs can penetrate cells and enter chloroplasts, where they are eventually degraded into CO_2_ and released. In contrast, He et al. and Zhang et al. did not detect the existence of GFNs in plants with TEM [9,39]. Interestingly, those two studies were conducted in soil, whereas all previous studies that detected GFNs in vivo were conducted using plants raised in growth medium. GO has been shown to exhibit varying degrees of affinity with different clay minerals [40], which would interfere with its absorption by plants. It is a reasonable conclusion that soil conditions are not conducive to the transport of GFNs into plants.

## 3. The Morphological Effects of GFNs on Plants

**Figure 1:**
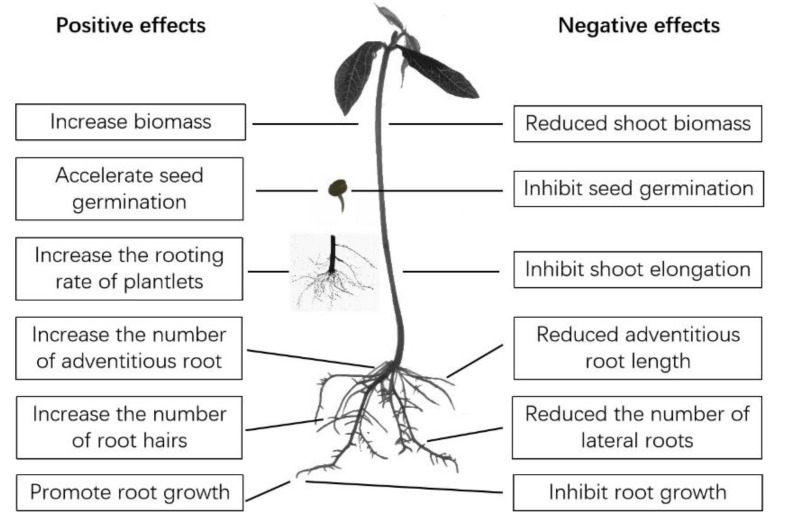
Diagram summarizing the effects of graphene-family nanomaterials (GFNs) on plant growth.

The impacts of GFNs on higher plants have been widely investigated, and both enhanced and inhibited growth have been reported for plants exposed to GFNs at various developmental stages (e.g., seed germination, root and shoot growth, and flowering) (Figure 1). The known positive and negative effects of GFNs on different plant species are shown in Table 1 and Table 2, respectively. The biological effects of GFNs seem to be closely related to variables such as the plant species and the physicochemical properties, concentration, exposure time, and application mode of the GFN [10].

The effects of GFNs on plant growth vary between different species and organs. Most studies have shown that GFN exposure can accelerate seed germination, the first step of plant development, because it promotes water absorption by seeds [9]. GFNs are reported to promote seed germination in spinach [9], chive [9], tomato [30], cotton [41], vinca [42], and wheat [43], but inhibit seed germination in rice [44]. As the organ of direct contact between plants and soil, roots play an important role in plant growth due to their absorption and fixation functions. In the seedling growth stage, GFNs have a large impact on plant root growth, which further affects the growth and development of above-ground organs. The growth of root and aerial organs, which ultimately determines plant biomass, was reportedly stimulated by GFNs in *Pinus tabuliformis* [39], *Aloe vera* [45], elm [46], maize [46,47], tomato [30,41], cotton [42], vinca [42], coriander [48], garlic [48], tobacco [49], and wheat [43,50,51] at appropriate concentrations. GFNs also have positive effects on vinca flower number [42], coriander and garlic flower growth [48], tobacco callus growth [49], and watermelon perimeter [52]. In contrast, GFNs inhibition of root and above-ground growth has been reported in species such as cabbage [53], tomato [53], red spinach [53], lettuce [53], rice [44,54,55], wheat [25,43,56], and *Brassica napus* [57,58,59]. 

Different GFNs may have varying effects on plant growth due to differences in physical and chemical properties. For instance, wheat seedling growth was significantly promoted by G-NH_2_ but inhibited by GO treatment [43]. Treatment with HGR rather than GO or RGO increased seed germination rate, root growth, and resistance to oxidative stress in wheat [51]. Compared to GO, treatment with lysine@graphene oxide (L-GO) and methionine@graphene oxide (M-GO) dramatically improved pearl millet growth, biomass, total protein content, photosynthetic pigment content, and yield under salt stress [60]. GO inhibited shoot and root growth at 100 and 250 mg/L, whereas RGO exhibited no obvious toxic morphological effects at the same concentrations [55]. 

**Table 1:** Positive effects of graphene-family nanomaterials (GFNs) in plants.

Plant Species.	Type of GFNs	Concentration (mg/L)	Exposure Duration	Growth Environment and Application Mode	Effects and Phenotype	Ref.
*Pinus tabuliformis* Carr.	GO	12.5, 25, and 50	180 days	Soil (irrigation)	Promote root growth with the best efficiency at 25 mg/L	[39]
*Aloe vera* L.	GO	10, 20, 50, and 100	120 days	Soil (irrigation)	Promote shoot and root growth with the best efficiency at 50 mg/L	[45]
elm (*Ulmus pumila* L.)	GO	50	60 days	Soil (irrigation)	Promote shoot and root growth	[46]
Spinach (*Spinacia oleracea* L.)	GO	50 and 200	30 days	Soil (irrigation)	Accelerate seed germination and increase the biomass at 50 mg/L	[9]
Chive (Allium schoenoprasum)	GO	50	45 days	Soil (irrigation)	Accelerate seed germination	[9]
Maize (*Zea mays*)	GO	20, 25, 50, and 100	30 days	Soil (irrigation)	Promote the growth of shoot and root	[47]
	GO	25, 50, 100, and 200	14 days	Soil (irrigation)	Promote the growth of shoot and root	[61]
Tomato (*Solanum lycopersicum* cv.)	GO	50, 100, and 200	30 days	Soil (irrigation)	Increase the root fresh weight and dry weight with the best efficiency at 100 mg/L	[41]
	Graphene	40	10 and 18 days	Water (hydroponic culture on cotton)	Accelerated seed germination at the initial 4 days; promote shoot and root elongation but reduce the total fresh weight after 18 days treatment	[30]
Cotton (*Gossypium hirsutum*)	Graphene	50 and 200	4 and 7 days	Growth medium and soil (irrigation)	Graphene could accelerate seed germination in growth medium and promote shoot and root growth in soil with the best efficiency at 200 mg/L	[42]
Vinca (*Catharanthus roseus*)	Graphene	50 and 200	4, 28, and 140 days	Growth medium and soil (irrigation)	Accelerate seed germination (4th day) in growth medium; promote shoot and root growth and amplify the biomass (28th day), increase flower number (140th day) in soil	[42]
Coriander (*Coriandrum sativam* L.), Garlic (*Allium sativum*)	GQD	200	3 h	Soil (Seed soaking)	Enhance the growth rate of leaf, root, shoot, and flower	[48]
Pearl millet (*Pennisetum glaucum* L.)	GO, L-GO, M-GO	20	165 days	Soil under saline conditions (foliar applications)	Increase the height and weight of plant under salt stress	[60]
Alfalfa (*Medicago sativa* L.)	Modified graphene	0.5%, 1%, and 2% (*w*/*w*).	40 days	Soil (mixed with the matrix)	Increase the fresh weight and dry weight of leaf at low concentration (0.5%), but decrease both at high concentration (2%)	[62]
Arabidopsis (*Arabidopsis thaliana* L.)	GO	0.1, 1, and 10	24 and 30 days	MS solid medium	Increase the flower bud number	[52]
Tobacco (*Nicotiana tabacum*)	FLG	50 and 100	30 days	MS solid medium	Promote callus cell culture growth at 50 mg/L,	[49]
	FLG	50 and 100	10 and 20 days	MS solid medium	Accelerate seed germination and promote root growth at 50 and 100 mg/L	[49]
	GO	20	20 and 35 days	Water (hydroponic culture on sponge)	Increase adventitious root number and root fresh weight (35th day), but inhibit seminal root growth (20th day)	[63]
Apple (*Malus domestica*)	GO	0.1, 1, and 10	40 days	1/2 MS solid medium	Increase the number of adventitious roots and the rooting rate at 0.1 and 1 mg/L	[64]
*Betula pubescens*	GO	0.00075, 0.0015, 0.003, 0.006, and 0.015	45 days	MS solid medium	Increase shoot length and leave number of microclone at 1.5 and 3 μg/L	[65]
Faba bean (*Vicia faba* L.)	GO	100, 200, 400, 800, and 1600	NA	Water (hydroponic culture on filter paper)	Accelerate seed germination and promote root growth at 800 mg/L	[66,67]
Wheat (*Triticum aestivum* L.)	SGO	50, 250, and 500	72 h	Hoagland solution (hydroponic culture)	Increase the relative growth rate of leaves at 50 and 250 mg/L	[50]
	G-NH_2_	125, 250, 500, 1000, and 2000	1, 3, and 9 days	Water (hydroponic culture)	Accelerate seed germination (1th day) and promote the growth of shoot and root (9th day) at 1 and 2 mg/L	[43]
	HGR	200	5 days	Water (hydroponic culture)	Accelerate age seed germination and increase root length, root number and root fresh weight	[51]
Watermelon (*Citrullus lanatus*)	GO	10	Once a week for 1 month	Inject the GO solution directly into the stem	Increase the perimeter of watermelon	[52]
Lettuce (*Lactuca sativa* L.)	GO	30 and 60	30 min	1/4 Hoagland nutrient solution (foliar applications)	Promote root growth	[68]

GO (graphene oxide), GQD (Graphene quantum dots), MS (Murashige and Skoog), FLG (few-layered graphene), RGO (reduced graphene oxide), L-GO (lysine@graphene oxide), M-GO (methionine@graphene oxide), SGO (sulfonated graphene oxide), G-NH_2_ (amine-functionalized graphene oxide), HGR (hydrated graphene ribbon), RGR (relative growth rate). NA means the related information is not provided.

**Table 2:** Negative effects of GFNs in plants.

Plant Species	Type of GFNs	Concentration (mg/L)	Exposure Duration	Growth Environment and Application Mode	Effects and Phenotype	Ref.
Cabbage (*Brassica oleracea* var. capitata), tomato (*Lycopersicon esculentum*), red spinach (*Amaranthus tricolor* L.), lettuce (*Lactuca sativa*)	Graphene	500, 1000, and 2000	20 days	Hoagland liquid medium (hydroponic culture)	Inhibit root and shoot growth	[53]
Rice (N22)	GO	2, 5, 7, and 10	1 and 16 days	Water (hydroponic culture on filter paper)	Inhibit seed germination and decrease the indexes of morphology (root length, stem length, adventitious number, and fresh weight) at 10 mg/L	[44]
Rice (9311)	Graphene	5, 50, 100, and 200	16 days	Water (hydroponic culture on filter paper)	Inhibit the indexes of morphology (root length, stem length, adventitious number, fresh weight and root cap ratio) at 100 and 200 mg/L	[54]
Rice (*Oryza. sativa* L.)	Graphene, GO and RGO	5, 50, 100, and 250	21 days	1/4 Hoagland liquid medium (hydroponic culture)	GO reduced shoot biomass and shoot elongation at 100 and 250 mg/L, while RGO exhibited no obvious effect	[55]
Wheat (*Triticum aestivum* L.)	GO	0.125, 0.25, 0.5, 1, and 2	1, 3, and 9 days	Water (hydroponic culture)	Inhibit the growth of shoot and root at 0.5, 1 and 2 g/L	[43]
	GO	40, 200, 400, 800, and 2000	7 and 15 days	Hoagland nutrient solution (hydroponic culture)	Inhibit shoot and root growth at 0.8 and 2 g/L	[25]
	Graphene	500, 1000, and 2000	10 days	Water (hydroponic culture)	Inhibit root and shoot growth	[56]
*Brassica napus* L.	GO	5 and 25	10 days	Water solution on sponge	Inhibit root growth at 25 mg/L	[57,58]
	GO	0, 10, 25, 50, and 100	15 days		Inhibit root growth, root fresh weight, and adventitious root number at 25, 50, and 100 mg/L	[59]
faba bean (*Vicia faba* L.)	GO	100, 200, 400, 800, and 1600	NA	Water (hydroponic culture)	Inhibit seed germination and root growth at 0.1, 0.2, and 1.6 g/L	[66,67]
Apple (*Malus domestica*)	GO	0.1, 1, and 10	40 days	1/2 MS solid medium	Inhibit the adventitious root length and the number of lateral roots	[64]
*Betula pubescens*	GO	0.00075, 0.0015, 0.003, 0.006, and 0.015	45 days	MS solid medium	Reduce shoot height of microclones at 15 μg/L	[65]

GO (graphene oxide), RGO (reduced graphene oxide), MS (Murashige and Skoog).

Similar to most growth-regulating substances, GFNs have concentration-dependent effects on plant growth (whether activating or inhibiting), and therefore an optimal concentration exists for inducing such effects. For example, the optimal concentrations of GO for promoting the growth of *Pinus tabuliformis, Aloe vera*, and tomato were 25, 50, and 100 mg/L, respectively [39,41,45]. Some studies have demonstrated hormetic biological effects of GFNs, meaning that the response is promoted at low GFN concentrations but inhibited at high concentrations. For example, Ren et al. showed a hormetic response of plant growth to SGO [69]; in another study, 1.5 and 3 µg/L GO increased shoot length and leaf number of *Betula pubescens* microclones, whereas 15 µg/L reduced shoot height [65].

There is a lag time between application of a substance and the biological effect, and different exposure times often correspond to different development stages. Thus, the biological effects of GFNs often differ with changes in exposure time. For example, plant incubation with graphene (250 - 1500 mg/L) for 24 h or 48 h induced no obvious alterations in leaf growth, but exposure for 30 d significantly reduced shoot biomass [70]. Application of 0.1 mg/L graphene to arabidopsis increased leaf area more significantly over 10 d compared to 5 d [57,60]. These results suggest that both short-term and long-term effects of GFNs should be considered when evaluating their biological effects in the following research.

At present, the primary GFN application methods are soil irrigation, addition to solid or liquid medium, and direct injection in vivo. Addition to aqueous solutions or growth media is a widely used strategy in research because the methods are simple, result in a high absorption rate, and include few interference factors. However, an increasing number of experiments have been conducted with soil treatments in recent years because these better mimic natural growth conditions of plants. There have also been some reports of foliar applications and direct injection. Foliar applications of 30 mg/L GO significantly promoted root growth and improved nutritional quality of lettuce [68]. This represents a promising result for GFNs in foliar fertilizer development. Although GO injection into aloe leaves did not directly promote leaf or root growth [45], injection of GO into the watermelon stem significantly increased the fruit perimeter [52]. This is a promising strategy for using GFNs precisely at the lowest possible concentration to minimize environmental release and should therefore be tested with other GFNs and plant species.

**Figure 2:**
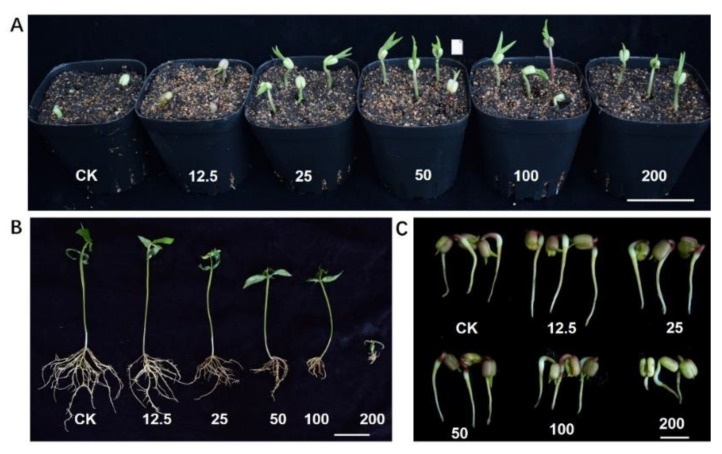
Effects of GO treatment on mung bean seedlings grown in different media. (**A**) Representative image of 5-day-old mung beans planted in soil. Scale bar = 5 cm. (**B**) Representative image of 10-day-old mung beans planted in solid MS medium. Scale bar = 5 cm. (**C**) Representative image of 3-day-old mung beans grown in water solution. Scale bar = 1 cm. Plants were treated with GO to a final concentration of 0, 12.5, 25, 50, 100, or 200 mg/L.

GFNs have almost always been reported to show positive effects for plants grown in soil by irrigation and negative effects for plants grown in aqueous solution or medium. We hypothesize that the environment in which plants grow is an important factor affecting the biological effects of GFNs. To test this hypothesis, we grew mung beans in soil, Murashige and Skoog (MS) solid medium, and water solution with 15–200 mg/L GO. The phenotypic analysis revealed that irrigation with GO solution accelerated seed germination and promoted shoot growth for mung beans planted in soil (Figure 2A). In contrast, GO inhibited shoot and root growth in a concentration-dependent manner for mung beans grown in solid MS medium (Figure 2B). For plants grown in the water solution, GO treatment promoted germination at low concentrations (12.5–50 mg/L) but exhibited obviously toxic effects at higher concentration (>100 mg/L) (Figure 2C).

The mechanisms underlying the variable biological effects of GFNs on plants grown in different media are unknown. Intuitively, water and mineral nutrients in aqueous and solid media are always abundant and readily available to plants, and GFNs may be less able to play their roles in such an environment. Compared with aqueous solution and solid medium, soil is a more complex and dynamic environment. A number of recent studies may shed light on this phenomenon. Graphene was shown to influence the concentrations of the major ions in soil as follows: sulfate > phosphate > ammonia > nitrate [71]. The oxygen-containing functional groups in GO confer excellent hydrophilicity, and it thus may function to improve the water supply by better collecting and retaining moisture in the soil [9]. As nano-carbons, GFNs may function in enhancing soil aggregation and reducing nutrient loss [41]. In addition, GFNs may affect plant growth by altering soil enzyme activity and microbial community [72]. GFN treatment decreases the activity of some soil enzymes, such as xylosidase, 1,4-β-N-acetyl glucosaminidase, and phosphatase [73], while increasing the activity of others, such as invertase, protease, catalase, and urease [74]. Although in vitro experiments showed that GFN treatment had a toxic effect on the soil microbial community [75], another study found that GO/GN application to the soil increased the diversity of the microbial community [74]. Compared with the mimic aquatic environment, GFN was shown to be much more retained in soil [40,76]. In addition, although high concentrations of GFNs can be toxic to plants, some substances in soil (such as humic acid) are natural antidotes [32]. 

## 4. The Physiological and Biochemical Effects of GFNs on Plants

Studies into the effects of GFNs on plant physiology and biochemistry have mainly focused on oxidative stress-related parameters, photosynthesis-related parameters, ion leakage, and water and nutrient content. Parameters related to oxidative stress include accumulation of reactive oxygen species (ROS); activity of antioxidant enzymes, such as peroxidase (POD), superoxide dismutase (SOD), and catalase (CAT); and levels of oxidized glutathione (GSSG) and malondialdehyde (MDA). Parameters related to photosynthesis include chlorophyll content, photosynthetic rate, transpiration rate, water use efficiency, and stomatal opening. 

In general, the negative effects of GFNs reported are increased ROS levels, decreases in antioxidant enzyme activity and photosynthesis-related parameters, increases in MDA and oxidized glutathione, elevated ion leakage, and alterations in moisture and nutrient content [31,53,66,67,77]. In contrast, the positive effects of GFNs tend to be lower ROS levels, enhanced antioxidant enzyme activity and photosynthesis-related parameters, and lower MDA content [51,60,63,64]. However, some studies show results that are not consistent with those discussed above. For example, although GO treatment promoted aloe vera growth, MDA levels were increased [45]. This suggests that there may be multiple mechanisms by which GFNs affect plant growth. We have proposed that GO treatment may mimic a weak stress signal, enhancing root growth and the plant’s ability to cope with the external environment [39]. 

Plant hormones, including auxin, cytokinin, abscisic acid (ABA), ethylene, and gibberellin (GA) are important trace substances that regulate plant growth. Changes in levels of these hormones often have a significant effect on the development of plant organs [78]. Studies have shown that positive effects of GO treatment are associated with an increase in IAA levels [41], whereas negative effects are accompanied by an increase in ABA and a decrease in IAA content [58,59]. 

## 5. The Cytological Effects of GFNs on Plants

Several forms of microscopy have been widely used to detect the cytological effects of GFN treatment. Because GFNs have a lamellar structure, cell membranes are vulnerable to mechanical damage by the sharp edges [79,80]. Optical microscopy has shown that stem thickening caused by GO treatment was mainly the result of an increase in cortical cell number [41] and that GO treatment resulted in strong disintegration and loss of epidermal and cortical cells in wheat root [43]. SEM images showed physical damage in wheat root treated with 1000 mg/L GO [81]. In addition, TEM has been used to observe plant ultrastructure, and several such studies have reported a lack of significant changes in cell ultrastructure in response to GFN treatment [9,43]. However, a large number of TEM observations have shown that accumulation of GFNs can lead to destruction of the vacuolar structure and cell membrane, increases in lysosome number, changes in chloroplast shape and the number of starch granules accumulated in chloroplasts, and even serious damage to structures, such as the nucleus [25,27,32,39,77].

## 6. Differential Gene Expression in Plants Treated with GFNs

Although the effects of GFNs on morphological, physiological, and cytological phenotypes in plants are well-established, little is known regarding responses at the gene expression level. Analysis of genes affected by GFN treatment not only provides clues for understanding functional mechanisms, but also has significance for effective harnessing of GFNs. First, affected genes could be used as molecular markers to identify the most suitable species and application modes for a given treatment quickly and accurately. Second, identification of affected functional genes would offer the opportunity to genetically engineer plants to mimic the effects of GFN treatment. In recent years, a large number of studies into plant gene expression after GFN treatment have provided opportunities to understand the effects of GFNs at the molecular level. The known differentially expressed genes (DEGs) in response to GFN treatment are shown in Table 3.

At present, nearly all studies on plant gene expression in response to GFN treatment are based on plant roots, and GFN treatment is likely to affect expression of genes that regulate root growth. For example, it has been reported that DEGs in plants treated with GFNs include *Adventitious Rooting Related Oxygenase 1* (*ARRO1*), *Transparent Testa Glabra 1* (*TTG1*), and *Auxin Response Factor 19* (*ARF19*) in apples [64]; *SlExt1* and *Constitutive Triple Response 1* (*LeCTR1*) in tomato [41]; and *BRN2*, *NAC2*, *MYB93*, and *Phytochrome-Interacting Factor 3 (PIF3)* in maize [61]. These are all candidate genes for promoting root growth. 

Transcription factors often play an important role in controlling plant growth by binding to and regulating the expression of a large number of other genes [82,83]. The expression of many transcription factors is altered after GFNs treatment, although their functions have not been accurately identified. Such transcription factors include *AP2-EREBP*, *MYB30 isoform X1*, *MYB6*, *MYB8*, *WRKY51*, *bHLH94*, *EMB1444*, *NAC32*, *Jungbrunnen 1 (JUB1), Wrinkled 1 (WRI1)*, *WRKY45, MADS-box transcription factor 26*, *NAC7,* and *ERF020* in maize [47,61] and *MYB3*, *MYB2*, *MYB4, NAC22*, *bHLH148*, *Sin3A Associated Protein 18* (*SAP18*), *Galactinol Synthase* (*GOLS*), and *NF-X-Like 1 (NFX1)* in *Pinus tabulaeformis* [39].

At present, hormone-related genes are the most abundant, biologically important, and well-understood genes controlling plant growth [84,85]. Genes reported in the literature to be regulated by GFNs include those involved in hormone synthesis, transport, signal transduction, and degradation. Hormone synthesis genes affected by GFN treatment include *SPINDLY* (*SPY*), *RGA*, *GAMYB*, *GA3*, and *GA5* for GA; *9-cis-epoxycarotenoid dioxygenase* (*NCED*), *Zeaxanthin Epoxidase* (*ZEP*), and *Abscisic acid Aldehyde Oxidase* (*AAO*) for ABA; *Cytokinin dehydrogenases* (*CKX1*, *CKX7*, *CKX5*, *CKX6*), and *tRNA isopentenyltransferases* (*IPT2*, *IPT3*, *IPT5*, *IPT7*) for cytokinin; *Brassinosteroid Insensitive 1-Associated Receptor Kinase 1* (*BAK1*), *Steroid 5-alpha-Reductase* (*DET2*), and *CPD* for brassinosteroids (BRs); *1-Aminocyclopropane-1-Carboxylic acid Synthase 2* (*ACS2*) for ethylene; and *Cam-Binding Protein 60-like G* (*CBP60*), *Systemic Acquired Resistance-Deficient 1* (*SARD1*), and *Isochorismate Synthase 1* (*ICS*) for salicylic acid (SA) [57,58]. DEGs associated with hormone transport include the auxin transporter genes *PIN-FORMED 7* (*PIN7*)*, ATP-Binding Cassette Subfamily B* (*ABCB1*), *LAX2,* and *LAX3* [64]. DEGs associated with hormone signal transduction include auxin-regulated transcription factors (*ARFs*, *IAAs*, *ABP20*, and *NAC71*); cytokinin downstream genes *ARR3*; the GA signaling pathway genes *MYBAS2*, *gibberellin responsive 1*, *gibberellin receptor GID1L2*, and *Chitin-Inducible Gibberellin-Responsive Protein 2* (*CIGR2*); the ABA receptor genes *Pyrabactin Resistance 1-Like* (*PYL2*, *PYL3*, and *PYL4*); the ABA-regulated genes *NAC2* and *WRKY24*; the jasmonic acid (JA)-regulated genes *TIFY10b* and *WRKY50*; the cytokinin response regulator *ARR3*; and the ethylene regulated *Ethylene-Responsive Transcription Factors (ERF2*, *ERF14*, *ERF34*, *ERF8*, *ERF12)* [47,61,63,64]. In addition, the expression of *Gibberellin 20 oxidase 2* and *Cytokinin Oxidase1*, which code key enzymes for GA and cytokinin degradation respectively, is upregulated by GO treatment [47,86]. 

GFNs also affect the expression of a large number of stress-related genes in plants, such as *Pyrroline-5-carboxylate Reductase* (*P5CR*), *Glycogen Synthase Kinase 3* (*GSK3*), and *Small Auxin Up-regulated RNA 41* (*SAUR41*) in *Pinus tabuliformis* [39]. In addition, GFN treatment altered the abundance of nitrogen and potassium metabolism genes, including *GLN6*, *nrt2*, *nrt2.2*, *Potassium transporter 20, 21* (*HAK20, HAK21*), and *kup1* [47]. Lastly, chloroplast photosystems may be affected by the altered expression of *psaA*, *psaB*, *psbD*, and *psbA*, which encode reaction center core proteins in response to GO treatment of wheat [50].

GFNs have been reported to promote the uptake of water and minerals in plants [9,87,88], and we sought to investigate the effects of GFNs on genes encoding membrane transport systems in the literature. Other than the auxin transporter genes mentioned above, nothing else was found. In fact, the expression of auxin transporter genes was regulated by auxin feedback [89], which means the alteration in these genes could be an indirect effect of GFN. We tend to think that the effects of GFNs on root absorption capacity may be independent of the expression of membrane system genes.

## 7. Conclusions

Few substances are known to exhibit such varied and antagonistic effects on plants as GFNs, and it is therefore difficult to derive a unified description of GFN mechanisms of action from the current literature. The diversity of effects attributed to GFNs treatment in different studies is caused by differences in plant species, the physicochemical properties of GFNs, concentration, exposure time, and application mode. Among these factors, we tend to think that the physical and chemical properties of GFN may be the most important reason for this contrast. After all, the GFNs used in different studies often come from different sources, and small changes in their modification groups often lead to dramatic changes in its function. The physical and chemical properties of GFNs are easily affected by the substances with which they come into contact. In the future, it should be possible to categorize GFNs by specific functions for uniform use in research and functional applications. We have proposed the creation of GFNs standards as soon as possible. In the absence of a single standard, comparative studies of the biological effects of various types of GFNs under the same conditions may make our conclusions more objective

Based on the available literature, it seems likely that GFNs can be absorbed by plant roots and transported to above-ground organs and that GFNs can cross cell membranes and enter organelles. In general, current understanding of the biological effects of GFN treatment is centered on oxidative stress, cell membrane systems, root growth/absorption capacity, and photosynthesis. GFNs may also indirectly affect plant growth by altering soil enzyme activity and microbial communities. Genes with altered expression in response to GFN treatment primarily include growth regulatory factors, transcription factors, hormone-related genes, nutrition metabolism-related genes, stress-related genes, and those encoding chloroplast proteins. However, the roles of these genes in responding to GFN treatment require further study.

## Figures and Tables

**Table 3:** Differentially expressed genes in response to GFNs treatment in plants.

Classification	Species and Tissue	Gene	Regulation	Treatment	Ref.
Root growth related	Apple (*Malus domestica*) root	*Adventitious Rooting Related Oxygenase 1* (*ARRO1*), *Transparent Testa Glabra 1* (*TTG1*), *Auxin Response Factor 19* (*ARF19*)	Up for 0.1 mg/L GO Down for 1 and 10 mg/L GO		[64]
	Tomato (*Solanum lycopersicum* cv.) roots	*SlExt1, Constitutive Triple Response 1* (*LeCTR1*)	Up	50 and 100 mg/L GO	[41]
	Maize (*Zea mays*) roots	*BRN2*	Up	50 mg/L GO	[61]
		*NAC2, MYB93, Phytochrome-Interacting Factor 3* (*PIF3*)	Down		
Transcriptional factors	Maize (*Zea mays*) roots	*AP2-EREBP*, *MYB30 isoform X1*, *MYB6*, *MYB8*, *WRKY51*, *bHLH94*, *EMB1444*, *NAC32*, *Jungbrunnen 1* (*JUB1*), *Wrinkled 1* (*WRI1)*	Up	50 mg/L GO	[47,61]
		*WRKY45*, *MADS-box transcription factor 26*, *NAC7*, *ERF020*	Down		
	*Pinus tabuliformis* Carr. roots	*MYB3*, *MYB2*, *MYB4*, *NAC22*, *bHLH148*, *Sin3A Associated Protein 18* (*SAP18*), *Galactinol Synthase* (*GOLS*), *NF-X-Like 1* (*NFX1*)	Up	25 mg/L GO	[39]
Auxin related	Apple (*Malus domestica*) roots	*PIN-FORMED 7 (PIN7), ATP-Binding Cassette Subfamily B* (*ABCB1*)*, LAX3*	Down	0.1, 1 and 10 mg/L GO	[64]
		*LAX2*	Up		
	Tomato (*Solanum* *lycopersicum* cv.) roots	*SlIAA3*	Down	50 and 100 mg/L GO	[41]
	*Brassica napus* L. seedling	*Auxin Response Factor 2, 8* (*ARF2*, *ARF8*), *IAA2*, *IAA3*	Up	25 and 50 mg/L GO	[57,58,59]
		*IAA4*, *IAA7*	Up for 25 mg/L GO Down for 50 mg/L GO		
	Maize (*Zea* *mays*) roots	*IAA9, ABP20 precursor*, *ABP20*	Up	50 mg/L GO	[47,61]
		*Auxin Response Factor 11* (*ARF11*), *PIN5c*, *NAC71*, *IAA24*	Down		
	Tobacco (*Solanum* *lycopersicum* cv.) roots	*IAA3*, *IAA4*, *IAA7*, *Auxin Response Factor 2*, *8* (*ARF2*, *ARF8*)	Up	20 mg/L	[63]
Cytokinin related	Apple (*Malus domestica*) roots	*ARR3*	Down	0.1 mg/L	[64]
	*Brassica napus* L. seedling	*Cytokinin dehydrogenase 1*, *7* (*CKX1*, *CKX7*), *tRNA isopentenyltransferase 2*, *3*, *5*, *7* (*IPT2*, *IPT3*, *IPT5*, *IPT7*)	Up	25 mg/L GO	[57,58]
		*Cytokinin dehydrogenase 5*, *6* (*CKX5*, *CKX6*)	Down		
	Maize (*Zea mays*) roots	*Cytokinin Oxidase1*, *Cytokinin-N-glucosyltransferase 1*, *cytokinin-O-glucosyltransferase 2*	Up	50 mg/L GO	[47]
GA related	*Brassica napus* L. seedling	*SPINDLY* (*SPY*), *RGA*, *GAMYB*, *GA3*, *GA5*	Up	25 mg/L GO	[57,58]
	Maize (*Zea mays*) roots	*Gibberellin 20 oxidase 2*, *gibberellin receptor GID1L2*, *gibberellin responsive 1*	Up	50 mg/L GO	[47]
		*Chitin-Inducible Gibberellin-Responsive Protein 2* (*CIGR2*), *MYBAS2*	Down		[61]
ABA related	*Pinus tabuliformis* Carr. root	*Pyrabactin Resistance 1-Like 2*, *3*, *4* (*PYL2*, *PYL3*, *PYL4*)	Down	25 mg/L GO	[39]
	*Brassica napus* L. seedling	*9-cis-epoxycarotenoid dioxygenase* (*NCED*), *Zeaxanthin Epoxidase* (*ZEP*)*, Abscisic acid Aldehyde Oxidase* (*AAO*)	Up	25 and 50 mg/L GO	[57,58,59]
	Maize (*Zea mays*) roots	*NAC2, WRKY24*	Down	50 mg/L GO	[61]
BR related	*Brassica napus* L. seedling	*Brassinosteroid Insensitive 1-Associated Receptor Kinase 1* (*BAK1*)	Up	25 mg/L GO	[57,58]
		*Steroid 5-alpha-Reductase* (*DET2*), *CPD*	Down		
SA related	*Brassica napus* L. seedling	*Cam-Binding Protein 60-like G* (*CBP60*)*,* *Systemic Acquired Resistance-Deficient 1* (*SARD1*)	Up	25 mg/L GO	[57,58]
		*Isochorismate Synthase 1* (*ICS*)	Down		
JA related	Maize (*Zea mays*) roots	*TIFY10b*, *WRKY50*	Down	50 mg/L GO	[61]
Ethylene related	*Brassica napus* L. seedling	*1-Aminocyclopropane-1-Carboxylic acid Synthase 2* (*ACS2*)	Up	25 mg/L GO	[57,58]
	Maize (*Zea mays*) roots	*Ethylene-Responsive Transcription Factor 2*, *14*, *34*, *8*, *12* (*ERF2*, *ERF14*, *ERF34*, *ERF8*, *ERF12*)	Down	50 mg/L GO	[61]
Nitrogen and potassium metabolism	Maize (*Zea mays*) roots	*GLN6*, *nrt2*, *nrt2.2*, *Potassium transporter 20*, *21* (*HAK20*, *HAK21*), *kup1*	Up	50 mg/L GO	[47]
Stress related	*Pinus tabuliformis* Carr. root	*Pyrroline-5-carboxylate Reductase (P5CR)*, *Small Auxin Up-regulated RNA 41* (*SAUR41*)	Up	25 mg/L GO	[39]
		*Glycogen Synthase Kinase 3* (*GSK3*)	Down		
Reaction center core proteins	Wheat (*Triticum* *aestivum* L.) leaf	*psaA*, *psaB*, *psbD*	Up	50, 250, and 500 mg/L GO	[50]
		*psbA*		50 and 250 mg/L GO	

## Data Availability

Not applicable.
